# Mortality amenable to healthcare in Latin American cities: a cross-sectional study examining between-country variation in amenable mortality and the role of urban metrics

**DOI:** 10.1093/ije/dyab137

**Published:** 2021-08-02

**Authors:** Pricila H Mullachery, Daniel A Rodriguez, J Jaime Miranda, Nancy López-Olmedo, Kevin Martinez-Folgar, Mauricio L Barreto, Ana V Diez Roux, Usama Bilal

**Affiliations:** 1 Urban Health Collaborative, Dornsife School of Public Health, Drexel University, Philadelphia, PA, USA; 2 Department of City and Regional Planning and Institute for Transportation Studies, University of California Berkeley, Berkeley, CA, USA; 3 School of Medicine, Universidad Peruana Cayetano Heredia, Lima, Peru; 4 CRONICAS Center of Excellence in Chronic Diseases, Universidad Peruana Cayetano Heredia, Lima, Peru; 5 Center for Population Health Research, National Institute of Public Health, Cuernavaca, Morelos, Mexico; 6 Department of Epidemiology and Biostatistics, Dornsife School of Public Health, Drexel University, Philadelphia, PA, USA; 7 Center for Data and Knowledge Integration for Health (CIDACS), Fundação Oswaldo Cruz, Bahia, Brazil

**Keywords:** Amenable mortality, urban health, Latin America

## Abstract

**Background:**

This study examined the variation in city-level amenable mortality, i.e. mortality due to conditions that can be mitigated in the presence of timely and effective healthcare, in 363 Latin American cities and measured associations between amenable-mortality rates and urban metrics.

**Methods:**

We used death records from 363 cities with populations of >100 000 people in nine Latin American countries from 2010 to 2016. We calculated sex-specific age-adjusted amenable-mortality rates per 100 000. We fitted multilevel linear models with cities nested within countries and estimated associations between amenable mortality and urban metrics, including population size and growth, fragmentation of urban development and socio-economic status.

**Results:**

Cities in Mexico, Colombia and Brazil had the highest rates of amenable mortality. Overall, >70% of the variability in amenable mortality was due to between-country heterogeneity. But for preventable amenable mortality, those for which the healthcare system can prevent new cases, most of the variability in rates occurred between cities within countries. Population size and fragmentation of urban development were associated with amenable mortality. Higher fragmentation of urban development was associated with lower amenable mortality in small cities and higher amenable mortality in large cities. Population growth and higher city-level socio-economic status were associated with lower amenable mortality.

**Conclusions:**

Most of the variability in amenable mortality in Latin American cities was due to between-county heterogeneity. However, urban metrics such as population size and growth, fragmentation of urban development and city-level socio-economic status may have a role in the distribution of amenable mortality across cities within countries.

Key Messages

- Rates of healthcare amenable mortality varied greatly in Latin American cities. A large share of the variation in amenable mortality for chronic and acute conditions was due to heterogeneity between countries.
- Cities in Mexico, Colombia and Brazil had the highest rates of amenable mortality, whereas cities in Argentina, Chile, Costa Rica and Panama had lower amenable-mortality rates compared with cities in other countries.
- Although a large share of the heterogeneity in amenable mortality in Latin American cities can be explained by country-level factors or by compositional differences across countries, city-level factors also play a role in driving rates of amenable mortality. In particular, high levels of urban-development fragmentation were associated with higher amenable mortality in large cities but lower amenable mortality in small cities. Cities with high socio-economic levels had relatively lower rates of amenable mortality.

## Introduction

Deaths amenable to healthcare are deaths attributed to conditions that can be mitigated in the presence of timely and effective healthcare.^1^ Examples include deaths due to ischaemic heart disease and tuberculosis among those aged <74 years and deaths due to diabetes among those aged <49 years.^2^ Although studies have examined amenable mortality in a number of high-income countries,^3^^,^^4^ less is known about amenable mortality in Latin American countries.^2^^,^^5^ In addition, little attention has been given to subnational variation, specifically variation in amenable mortality among cities within countries.

Over the past 30 years, Latin American countries have greatly expanded healthcare coverage for their citizens.^6^ Some countries, e.g. Brazil and Costa Rica, have created tax-financed universal healthcare systems where health services are available to all residents. Other countries, e.g. Argentina, Chile, Mexico and Peru, have expanded their healthcare coverage by subsidizing health insurance for the poor and uninsured, and pooling together funds from workers’ contributions and general taxes.^7^ As a result, countries in the region have greatly improved the coverage of health services and narrowed the gap in healthcare utilization.^7^^,^^8^ Despite this progress, issues around the quality of care received and care coordination for people with chronic conditions continue to be major barriers to improving population health.^2^^,^^9^ In addition, there is potential for large local variation in access to quality healthcare across geographies.^10^

In Latin America, as in low- and middle-income countries across other regions, a growing proportion of the population live in urban areas.^11^ The characteristics of the built environment in these cities may be determinant for access to quality healthcare.^10^^,^^12^ Historically, large cities in Latin America concentrate a significant portion of the countries’ healthcare resources, from infrastructure to human capital.^13–16^ Furthermore, the spatial configuration of cities may be associated with geographic barriers to healthcare access.^10^ Urban development that is discontinuous and spread out, i.e. urban-development fragmentation, is associated with increases in the cost of public services, traffic congestion and air pollution;^17^^,^^18^ this fragmentation may be an indicator of barriers to access to services in general, and healthcare services in particular. For example, urban-development fragmentation may limit geographic access among populations living in the peripheral areas of large urban centres, potentially reducing the benefits of living in a large city with relatively high availability of services but where services are distributed unevenly.

Socio-economic factors also play a role in access to healthcare;^19^ wealthier cities are more likely to concentrate healthcare resources as well as a large labour pool which, in turn, attracts populations from other areas of the country.^20^ On the other hand, rapid population growth could lead to increased demand for services for which the healthcare system is not prepared to provide. Examining the role of these urban features on amenable mortality may have the potential to inform policies to improve health outcomes among residents of Latin American cities and other low- and middle-income regions in the world.

The aims of this paper are: (i) to estimate the rates of mortality amenable to healthcare in 363 Latin American cities, including the share of city variability between and within countries; and (ii) to explore the relationships of urban metrics, including population size, population growth, urban-development fragmentation and city-level socio-economic status, with amenable-mortality rates.

## Methods

### Study setting

We used data from the Salud Urbana en America Latina (SALURBAL) project.^21^^,^^22^ This project has compiled and harmonized health and environmental data for 371 cities with populations of >100 000 people in 11 Latin American countries. Cities were defined as urban agglomerations of local administrative units that encompassed the urban extent or footprint of a city. For this analysis, we used data from 363 SALURBAL cities in nine Latin American countries (Argentina, Brazil, Chile, Colombia, Costa Rica, Mexico, Panama, Peru and El Salvador). We used data from 2012 to 2016 for all countries except El Salvador, for which we had data available from 2010 to 2014. The sources of mortality data and population denominators are listed in Supplementary Table S1 (available as Supplementary data at *IJE* online), whereas sources for all other data are available elsewhere.^23^ The SALURBAL study protocol was approved by the Drexel University Institutional Review Board with ID#1612005035.

### Outcome variables

Healthcare amenable deaths were defined using the codes recommended by Kruk and colleagues.^2^ This list of codes includes the conditions identified by Nolte and McKee^1^ and conditions included by Goal 3 of the Sustainable Developmental Goals (to ensure healthy lives and promote well-being for all at all ages) for which risk of death can be reduced by the use of personal healthcare. Amenable deaths were classified into three groups: (i) acute conditions that are treated or cured with episodic care, e.g. ischaemic heart disease and road injuries; (ii) chronic conditions requiring sustained care to either cure or prevent sequelae, e.g. diabetes and breast cancer; and (iii) conditions for which healthcare can prevent new cases, e.g. tuberculosis and cervical cancer (see the complete list of conditions in Supplementary Table S2, available as Supplementary data at *IJE* online).^2^ Population data for denominators were from population projections by age, sex and city, provided by local census bureaus.^22^ The main outcome of this study was sex-specific age-adjusted amenable mortality per 100 000, overall and by the three groups of amenable conditions, for each city, calculated using the direct method of standardization with the 2000–2025 WHO standard population.^24^

We corrected for the incomplete coverage of all death counts^25^ using an ensemble of death-distribution methods at the city level, stratified by sex. To address the potential violation of the assumption of no net migration for these methods, we used two approaches. First, we calculated completeness of death registration using three death-distribution methods that respond differentially to migration:^25^^,^^26^ (i) the generalized growth balance method (GGB), (ii) the synthetic extinct generations method (SEG) and (iii) the hybrid GGB-SEG method.^25^^,^^26^ Second, we estimated completeness at ages at which migration is lowest: (i) 30–65 years, as suggested by Hill;^27^ (ii) 50–70 years, as suggested by Murray;^26^ and (iii) best-fitting age bands^25^ using the R package Death Registration Coverage (DDM). We truncated all coverage estimates of >1 to 1 (assuming no overcounting) and averaged all nine estimates (three methods × three age bands) using the harmonic mean.^25^ More details about this correction are available elsewhere.^23^ We corrected death counts by dividing the number of deaths by the correction factor.

We also addressed deaths with an underlying cause coded as ill-defined diseases or injuries of ill-defined intent. We redistributed these ill-defined deaths to amenable deaths categories or non-amenable using a proportional redistribution, following the Global Health Estimates (GHE) method.^28^ Specifically, we fitted a multinomial model by country with age, sex and year as predictors, and obtained 100 draws of the redistributed cause of death from this model to acknowledge uncertainty in the redistribution.

Supplementary Figure S1 (available as Supplementary data at *IJE* online) shows the distribution of completeness and proportion of ill-defined deaths by city and country.

### Exposure variables

We used the following urban metrics as exposures: (i) the average city population in the study period; (ii) a measure of urban-development fragmentation, patch density, defined as the number of continuous areas of urban development per 100 square kilometres of land area; (iii) population growth defined as the percent change in population size between the first and last year for which mortality data were used in this analysis; and (iv) a composite index to proxy socio-economic status (SES) based on education attainment at the city level. This index was calculated by averaging the Z scores of two census-derived indicators: proportion of the population aged ≥25 years who had completed secondary education or above, and proportion of the population aged ≥25 years who had completed university education or above. The education categories were harmonized across countries.^22^ More details on the calculation of the patch density and the harmonization of the education variables across the countries can be found elsewhere.^22^

### Covariate adjustment

We used the percentage of the area of the city that is built, defined as the percentage of the total land area that is covered by urban patches.^29^ This adjustment was done to account for cities that may spread across areas that include largely unpopulated sections of land.

### Analytical approach

We first described the variability in outcomes, exposures and covariates by city size in tabular form. We calculated the age-standardized amenable-mortality rates by group (acute, chronic and preventable) across all redistributions and used the average rate in each city for descriptive purposes. For aim 1, to describe the variation in healthcare amenable mortality between and within countries, we examined amenable mortality by group, sex and country. To quantify the degree of variability between and within countries, we modelled the log of the sex-specific age-adjusted amenable-mortality rates using multilevel linear regression with cities nested within countries. We used the model below:

$$\log\left(y_{ij}\right)=\beta_{0}+\delta_{j}+\epsilon_{ij}\delta_{j}∼N\left(0,\tau^{2}\right)\epsilon_{ij}∼N\left(0,\sigma^{2}\right)$$

where *i* represents the cities within countries (*j*), *β*_0_ is the average amenable mortality (intercept), *δ*_*j*_ is the random effect for each country and ε_*ij*_ is the level-1 residual. *τ*^2^ and *σ*^2^ are the variance components that we used to calculated intra-class correlation coefficient (ICCs) that estimate the proportion of variation in amenable-mortality rates among countries (between-country variability) within the total variability [ICC = *τ*^2^/(*τ*^2^+ *σ*^2^)].

For aim 2, explore the relationships between urban metrics and amenable mortality, we regressed the log of mortality rates stratified by sex on all predictors simultaneously: population size on the log scale, population growth, patch density and city-level SES, adjusting for percentage of built-up area and country fixed effects. We also tested interactions between population size and the other predictors, i.e. patch density, population growth and SES index, to examine whether associations between these predictors and amenable mortality changed in cities with different population sizes. To facilitate the interpretation of coefficients, all variables were standardized by subtracting the mean and scaling by their standard deviation (SD). Models were run 100 times with the redistributed mortality rates for each city. Coefficients were pooled using Rubin's formula.^30^ To check for potential issues associated with the quality of the mortality data, we performed a sensitivity analysis using the same analytical strategy as described previously but excluding cities in El Salvador and Peru, given the lower levels of coverage of death counts in these countries. All analyses were performed using R version 4.0.2 and Stata 13.^31^ There was no patient or public involvement in either the design or the development of the study.

## Results

We analysed >8 million deaths over a 5-year period in 363 Latin American cities. Table 1 shows the median values for outcome, predictors and covariates. The median age-adjusted amenable-mortality rate was 493.3 per 100 000 (Q1–Q3 = 420.8–558.1) for women and 838.5 per 100 000 (Q1–Q3 = 713.9–946.5) for men. Cities with ≥5 million people had the lowest amenable-mortality rates. Among the 363 cities, the median population was 0.3 million. Supplementary Figure S2 (available as Supplementary data at *IJE* online) shows the relationship between amenable-mortality rate and population as a continuous variable in the log scale. The median 5-year population growth was 4.7% with larger growth in mid-sized cities. The median patch density was 29.5 patches per square kilometre and the median percentage built up was 3.5%. More-populated cities had generally higher patch density (higher fragmentation), percentage built up and SES compared with less-populated cities.

**Table 1.** Median outcomes, predictors and covariates in 363 Latin American cities by category of population size

Population size	Overall	100–250 K	≥250–500 K	≥500 K–1 M	≥1–5 M	≥5 M
# Cities	363	157	97	59	43	7
Amenable mortality rate (women)	493.3	495.7	503.7	482.4	493.3	426.6
	[420.8; 558.1]	[412.7; 569.1]	[421.5; 555.7]	[415.5; 542.4]	[439.2; 564.0]	[313.2; 533.4]
Amenable mortality rate (men)	838.5	846.2	840.9	838.5	831.5	671.7
	[713.9; 946.5]	[724.6; 969.8]	[681.4; 939.0]	[750.9; 943]	[715.8; 937.1]	[571.7; 852.2]
City size (million)	0.3	0.2	0.3	0.7	1.8	12.2
	[0.2; 0.6]	[0.1; 0.2]	[0.3; 0.4]	[0.6; 0.9]	[1.2; 3.1]	[8.6; 20.6]
City growth (%/5 years)	4.7	4.5	4.8	5.2	4.6	3.5
	[3.5; 6.4]	[3.3; 6.1]	[3.6; 6.7]	[4.1; 6.4]	[3.2; 6.2]	[2.9; 4.4]
Fragmentation (patches/km^2^)	29.5	23.3	24.7	35.4	61.1	56.7
	[11.9; 55.9]	[8.9; 41.1]	[10.1; 43]	[16.5; 58.9]	[40.2; 81.8]	[43.5; 91.5]
Percentage built up (%)	3.5	2.3	2.7	4.7	10.8	23.8
	[1.4; 6.6]	[0.9; 4.2]	[1.5; 5.8]	[2.5; 7.9]	[6.7; 13.1]	[19.4; 27.3]
Socioeconomic Index	–0.4	–1.0	–0.6	0.6	0.3	1.0
	[–1.3; 0.5]	[–1.7; 0.0]	[–1.3; 0.4]	[–0.7; 1.1]	[–0.2; 0.7]	[–0.3; 1.6]

Values are medians [Quartile 1; Quartile 3].

**Figure 1.**
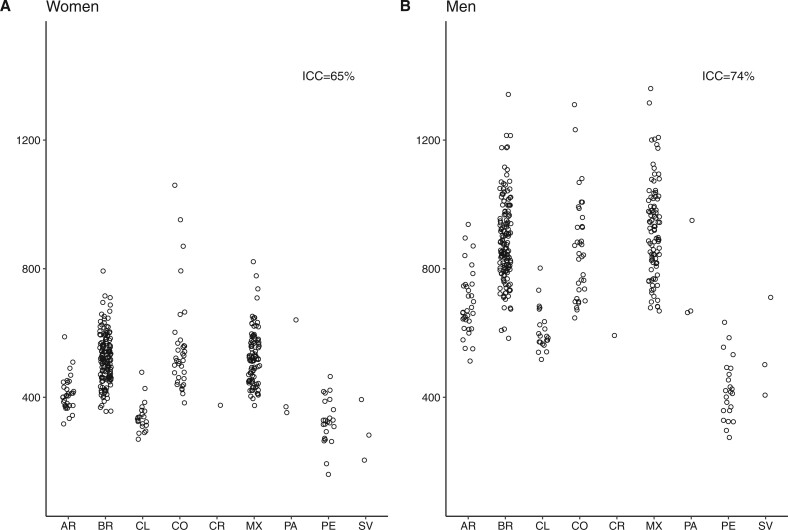
Amenable-mortality rates in 363 Latin American cities. AR, Argentina; BR, Brazil; CL, Chile; CO, Colombia; CR, Costa Rica; MX, Mexico; PA, Panama; PE, Peru; SV, El Salvador.

**Figure 2.**
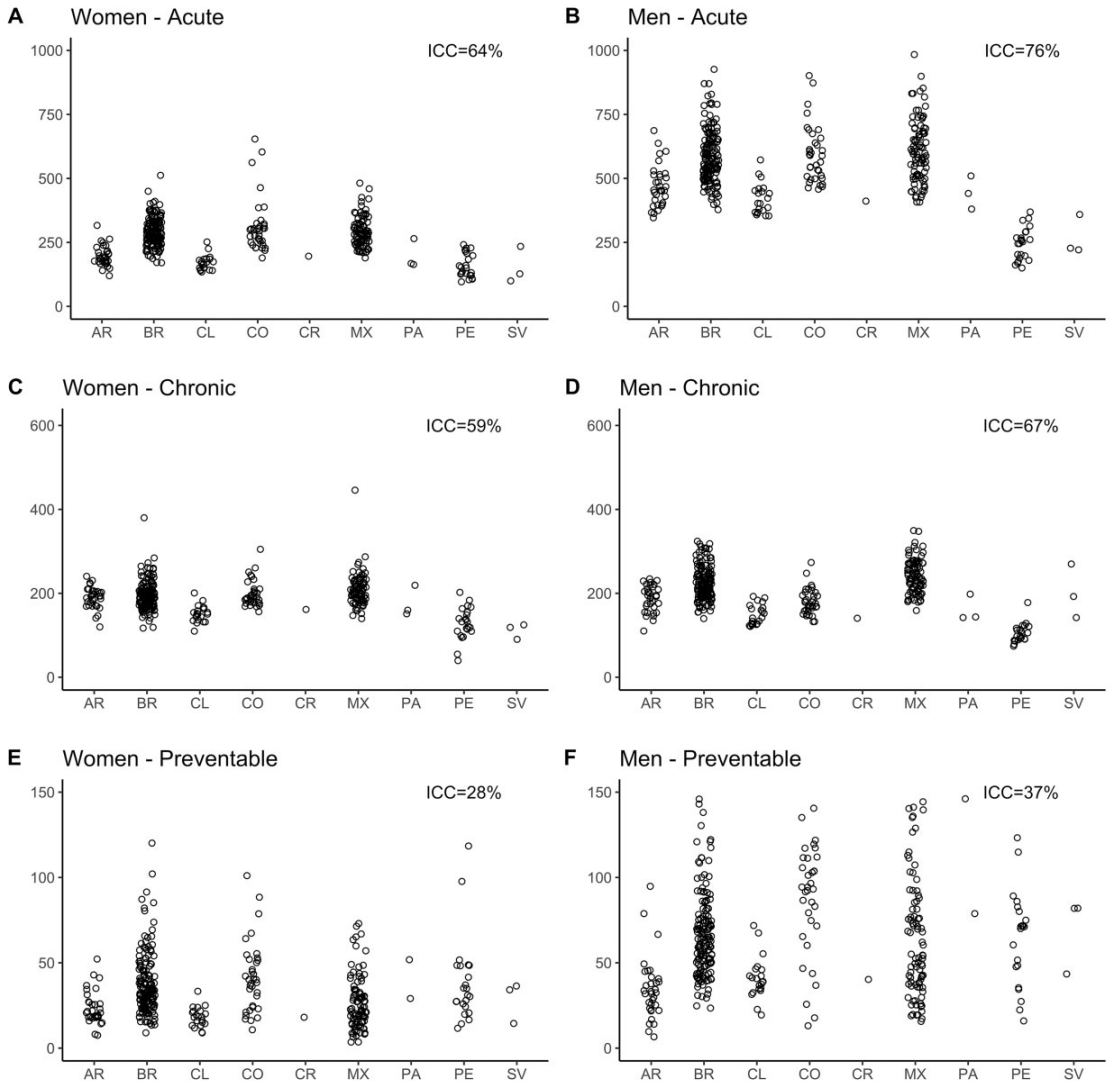
Amenable-mortality rates in 363 Latin American cities. AR, Argentina; BR, Brazil; CL, Chile; CO, Colombia; CR, Costa Rica; MX, Mexico; PA, Panama; PE, Peru; SV, El Salvador.

Figures 1 and 2 (and Supplementary Table S3, available as Supplementary data at *IJE* online) show amenable-mortality rates overall and for the three groups of amenable conditions: those requiring sustained care (chronic), those treated with episodic care (acute) and those for which access to healthcare can prevent incident cases (preventable) across countries for women and men. In general, median amenable-mortality rates were higher for men compared with women. For both men and women, Mexico, Colombia and Brazil had the highest amenable-mortality rates, ranging from 356 to 1059 per 100 000 in women and from 538 to 1360 in men. Argentina, Costa Rica, Panama and Chile generally had lower amenable rates, with rates ranging from 269 to 640 and 512 to 937 per 100 000 in women and men, respectively. Last, cities in El Salvador and Peru had the lowest amenable-mortality rates ranging from 160 to 464 and from 274 to 710 per 100 000 in women and men, respectively. This pattern of countries with high, intermediate and low mortality was similar for acute and chronic amenable-mortality rates. Acute and chronic amenable mortalities in Peru and El Salvador were particularly low. The pattern was different for preventable amenable mortality; Colombia, Panama and El Salvador had the highest preventable mortality rates among women and men. Argentina, Chile and Costa Rica had the lowest rates of preventable amenable mortality.

Figures 1 and 2 also show that a sizable proportion of the variation in amenable mortality due to acute conditions (ICC = 64% and 76%, for women and men, respectively) and chronic conditions (ICC = 59% and 67%) is due to between-country heterogeneity. For amenable mortality due to preventable causes, only 28% and 37% of the variability in rates, respectively for women and men, was due to between-country heterogeneity, meaning that most of the variability in preventable amenable mortality occurs between cities within countries.

**Figure 3.**
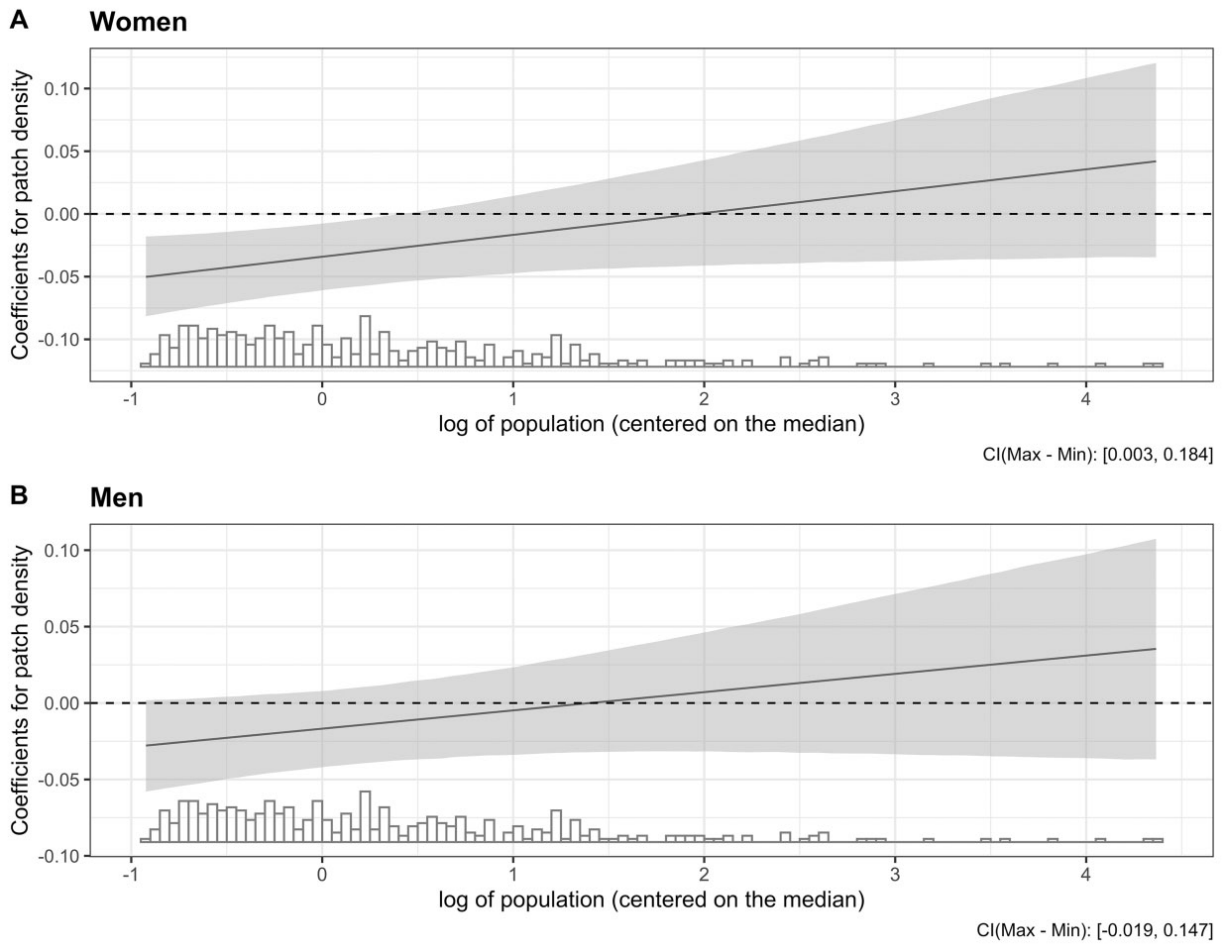
Predicted coefficients of association of fragmentation (patch density) with amenable mortality on population size derived from interactions in Table 2

Table 2 shows the adjusted associations between predictors and amenable mortality among women and men from the final model. We found an interaction between population size and patch density, and retained it in the final models. In average-sized cities (∼275 000), higher fragmentation was associated with lower amenable mortality: a 1-SD higher patch density was associated with 3.4% and 1.7% lower amenable mortality among women and men, respectively. However, the direction of the association between patch density and amenable mortality changed from negative to positive and became stronger as the population increased (Figure 3). Analogously, the association between city size and amenable mortality also differed at different levels of fragmentation. Among cities with average fragmentation, a larger population was associated with a lower amenable-mortality rate in women but higher amenable mortality in men; a 50% larger population in a city with the mean patch density was associated with a 0.6% lower [95% confidence interval (CI) –0.5% to 1.6%] and a 0.1% higher (95% CI –0.9% to 1.0%) amenable mortality among women and men, respectively. The association of city size and amenable mortality became more positive and stronger at higher levels of fragmentation.

**Table 2.** Percent differences in amenable mortality associated with urban metrics among women and men in 363 Latin American cities

	Women	Men
Population (% difference in mortality for a 50% difference in population in a city with the mean patch density)	–0.6%	0.1%
	[–0.5%, 1.6%]	[–0.9%, 1.0%]
Patch density (% difference in mortality for a 1-SD difference in patch density for a city with population 275 000)	–3.4%	–1.7%
	[–5.9%, –0.7%]	[–4.1%, 0.8%]
Population × patch density	0.02	0.01
	[0.000, 0.034]	[–0.004, 0.028]
Growth (% difference in mortality for a 1-SD difference in % growth)	–2.7%	–2.0%
	[–4.3%, –1.0%]	[–3.5%, –0.4%]
SEI (% difference in mortality for a 1-SD difference in the Socioeconomic Index)	–3.3%	–3.2%
	[–5.4%, –1.2%]	[–5.2%, –1.2%]

95% confidence intervals in brackets. Models were run 100 times with the redistributed mortality rates for each city and coefficients were pooled using Rubin's formula. Population size was centred at the median (∼275 000). Models were adjusted for % built up and country. SEI, Socioeconomic Index; SD, standard deviation.

A 1-SD higher SES index was associated with a 3.3% and 3.2% lower amenable mortality among women and men, respectively. Finally, a 1-SD higher population growth, measured as a percentage change from the first and the last year, was associated with 2.7% and 2.0% lower amenable mortality among women and men, respectively.

**Figure 4.**
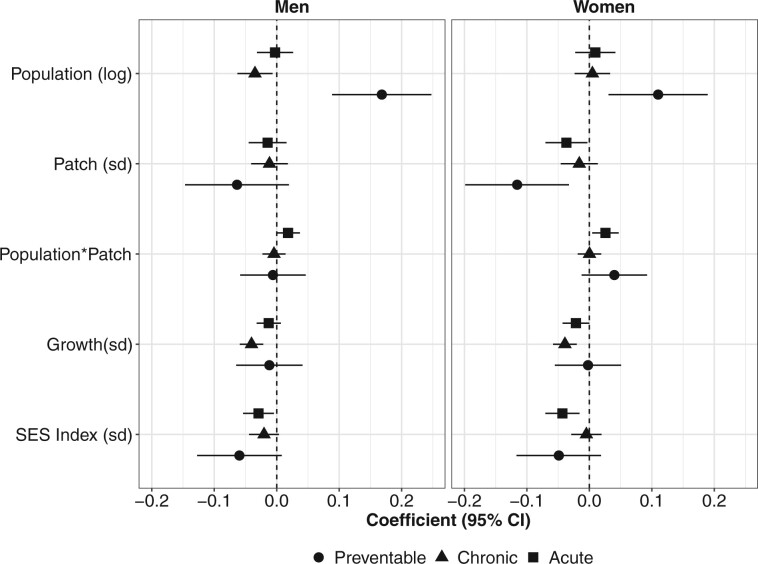
Estimates from final models by sex and groups of amenable mortality in 363 Latin American cities. SES, socio-economic status.

The groups of amenable mortality (chronic, acute and preventable) had generally similar patterns of association with predictors, with some important caveats (Figure 4). A larger population size was associated with a higher mortality rate by preventable conditions in both women and men, but with a lower chronic amenable-mortality rate in men. Positive population growth was associated with lower chronic amenable mortality, but not associated with acute or preventable amenable mortality. Last, the interaction between population size and fragmentation was observed for acute amenable mortality only.

The results from the sensitivity analyses excluding El Salvador and Peru showed estimates that were broadly consistent with the main analysis (Supplementary Figure S3, available as Supplementary data at *IJE* online).

## Discussion

Rates of healthcare amenable mortality varied greatly in Latin American cities, from <400 to >900 deaths per 100 000 people. More than two-thirds of the variation in amenable-mortality rates was due to between-country heterogeneity, whereas a third was due to heterogeneity between cities within countries. Cities in Mexico, Colombia and Brazil had the highest rates of amenable mortality due to acute and chronic conditions, whereas cities in Argentina, Chile and Panama had lower amenable-mortality rates compared with cities in other countries. Cites in El Salvador and Peru had lower amenable-mortality rates than most other countries but higher mortality rates due to preventable causes, those for which the healthcare system can prevent cases. We found that higher urban-development fragmentation was associated with lower amenable mortality in smaller cities but higher amenable mortality in larger cities. We also found that faster population growth and higher city-level SES were associated with lower amenable mortality.

A potential explanation for the large proportion of between-country variability in amenable mortality is related to differences in the healthcare systems across the countries. Latin American countries vary in the financing and organization of their healthcare systems.^6^ Argentina, Chile and Panama have the highest healthcare spending from government or compulsory contributory schemes (mainly social security), at ∼$1100 per capita in 2016 adjusted for purchase power parity (PPP).^32^ Brazil, Mexico and Colombia had relatively low healthcare expenditures coming from government/compulsory schemes, at ∼$500 per capita in Brazil and Mexico and $700 per capita in Colombia. Peru and El Salvador had the lowest healthcare expenditure, at ∼$400 per capita adjusted for PPP.^32^ Our amenable-mortality results are generally consistent with the pattern of healthcare spending across countries: the lower the healthcare spending, the more amenable mortality was observed. Peru and El Salvador are exceptions to this pattern, which may be explained by issues around the quality of vital-statistics systems in these countries. Although excluding cities from the two countries did not affect the overall associations, inferences about the amenable-mortality rates in these cities are limited by data-quality issues. Differences in behavioural factors across countries and cities may also play a role in the heterogeneity seen in amenable mortality. Heterogeneity in the prevalence of behavioural and cardiometabolic risk factors in Latin American cities^33–35^ is likely to lead to variations in amenable mortality above and beyond the role of healthcare systems.

Previous studies examining amenable mortality have focused on high-income countries and used different lists of causes of death to classify amenable mortality, precluding direct comparison with our results. A small number of studies have examined amenable mortality in low- and middle-income countries using adapted lists of amenable conditions. Kruk *et al.*^2^ estimated that in 2016 a total of 8.6 million deaths were amenable to healthcare in 137 countries. However, this paper applied a different methodology to calculate age-adjusted amenable-mortality rates and focused only on country-level metrics. Our study allowed the examination of variability in amenable mortality across cities as well as city-level predictors of amenable mortality.

For all amenable mortality, we found an interaction between patch density, a measure of urban-development fragmentation, and population size, such that higher fragmentation was associated with higher amenable mortality in larger cities (and, analogously, a larger city size was associated with higher amenable mortality in more fragmented cities). One potential explanation for this finding is that high urban-development fragmentation can represent geographic barriers for city residents living in peripheral areas of large urban centres.^10^ In smaller cities, however, higher fragmentation was associated with lower amenable mortality. Fragmentation may also mean that interstitial spaces, likely to be green,^36^ provide opportunities for mental restoration and physical activity. Indeed, fragmentation of urban development has been associated with decreased noise,^37^ lower heat-island effects^38^ and better air quality.^39^ Future studies should examine the associations of urban metrics with specific causes of deaths to empirically test these potential explanations in large and small cities. An interesting finding was that population size was associated with higher amenable mortality due to preventable conditions regardless of fragmentation. This could be related to the higher prevalence of some causes of preventable amenable mortality (e.g. tuberculosis) in denser areas.^40^ Finally, population growth, which was associated with lower amenable mortality, may be a proxy for thriving cities with a large share of the workforce formally employed.^23^

To our knowledge, this is the first study to examine the associations of urban-development fragmentation and city size with amenable mortality. In our sample of cities, fragmentation was much higher in cities with ≥1 million people. This may indicate the challenge of managing urban growth in larger cities effectively. Larger cities generally have lower mortality rates but important variations across neighbourhoods may exist.^29^ Further examination of mortality variation across neighbourhoods within cities is needed to identify areas with relatively high and low mortality, and whether patterns of amenable mortality within cities are associated with access to healthcare. Our results also showed that cities with higher SES (proxied by levels of educational achievement) had on average lower amenable mortality. Educational attainment is known to predict access to important resources and affect several health outcomes via multiple mechanisms.^41^ One potential mechanism is higher education leading to access to high-quality healthcare, which can mitigate death and disability. Future studies should examine causal mechanisms in specific groups or amenable conditions.

A limitation of our study is the use of administrative units that contained the urban footprint of the city, which in some countries led to large units of analysis that included some undeveloped areas. Even though we adjusted for the percentage of the area that is built, this may have led to misspecification of the urban constructs in our analyses. A number of other factors that may be relevant predictors of healthcare amenable mortality, including those related to healthcare access and quality,^2^ were not included in our analysis. In addition, we did not adjust for cardiometabolic and behavioural factors that contribute to the overall burden of disease, such as smoking and obesity, given the challenges involved in obtaining city-level prevalence rates of these factors across several countries. Under-reporting of deaths and ill-defined codes are still important issues limiting the quality of the mortality data worldwide and vary considerably across countries. To address ill-defined codes, we used proportional redistribution by age, sex, country and year, following the GHE method.^28^ We chose this approach due to its relative simplicity, as opposed to the more complex^42^ Global Burden of Disease (GBD) method.^43^ The quality of mortality estimates has been shown to depend on the quality of vital registration,^44^ which according to GBD is very high in the countries in which 337 of our 363 cities are located.^43^ However, despite these steps, we cannot completely rule out the possibility of bias resulting from these issues. Future studies should consider leveraging new methodologies to address completeness, including model-based estimates of completeness^45^ and more detailed examination of ill-defined codes.^46^ We believe that the use of mortality data at the subnational level can help to identify and address issues of quality and completeness, and help to advocate for improved data quality.^23^^,^^47^

In conclusion, we found that sex-specific age-adjusted amenable-mortality rates vary greatly across 363 cities of >100 000 people in nine Latin American countries. However, most of the total variability in amenable mortality was due to between-country heterogeneity, likely due to differences in macro determinants of health across countries as well as country-level healthcare-system factors. Urban metrics such as population size, population growth, fragmentation of urban development and city-level SES may have a role in the occurrence of amenable mortality. Specifically, high levels of urban fragmentation are a potential indicator of geographical barriers to healthcare in large urban centres, which in turn may lead to high amenable mortality in large cities with high fragmentation. Understanding the links between urban metrics and city residents’ health may provide valuable evidence to urban planners and public health practitioners looking to plan healthier cities and improve access to healthcare.

## Supplementary data

Supplementary data are available at *IJE* online.

## Ethics approval

The SALURBAL study protocol was approved by the Drexel University Institutional Review Board with ID#1612005035.

## Funding

This project was supported by the Wellcome Trust initiative, ‘Our Planet, Our Health’ [Grant 205177/Z/16/Z] and by the Office of the Director of the National Institutes of Health under award number DP5OD26429. The funding agencies had no involvement in the study design; in the data collection, analyses or interpretation of the data; in the writing of this work; or in the decision to submit the manuscript for publication.

## Data availability

Mortality data for Brazil, Chile, Colombia and Mexico were downloaded from publicly available repositories from statistical agencies in each country. Mortality data for Argentina, Costa Rica, El Salvador, Panama and Peru were obtained directly from statistical agencies in each country. A link to these agency websites can be accessed via https://drexel.edu/lac/data-evidence/data-acknowledgements/. Sources of data are provided with this paper.

## Supplementary Material

dyab137_Supplementary_Data
