# Supplementary material for: Mortality amenable to healthcare in Latin American cities: a cross-sectional study examining between-country variation in amenable mortality and the role of urban metrics
Source: Int J Epidemiol. Author manuscript; Available in PMC 2022 Feb 28. (PMC8856009; doi:10.1093/ije/dyab137)
Supplement: Supplementary Data [file EMS133515-supplement-Supplementary_Data.docx]

**Supplementary material**

Table S1: Data sources for mortality in SALURBAL countries and completeness of vital registration records.

The SALURBAL team obtained information on mortality records from Argentina, Brazil, Chile, Colombia, Costa Rica, El Salvador, Mexico, Panama, and Peru. Data was sent by each country team in a de-identified database containing records for each individual death, which included municipality of residence, age at death, and cause of death using the International Classification of Diseases, version 10 (ICD-10). Data for population denominators were obtained from various sources in each country. See data sources for mortality and population data below:

| **Country** | **Mortality data source** |
| --- | --- |
| Argentina | Dirección de Estadística e Información de Salud (DEIS) |
| Brazil | Ministério da Saúde, Departamento de Informática do SUS (DATASUS) |
| Chile | Departamento de Estadísticas e Información de Salud (DEIS) |
| Colombia | Departamento Administrativo Nacional de Estadísticas (DANE). |
| Costa Rica | Instituto Nacional de Estadística y Censos Costa Rica (INEC). |
| El Salvador | Dirección General de Estadística y Censos (DIGESTYC). |
| Mexico | Instituto Nacional de Estadística, Geografía e Informática (INEGI). |
| Panama | Instituto Nacional de Estadística y Censos (INEC). |
| Peru | Instituto Nacional de Estadística e Informática (INEI) |
| **Country** | **Population data source** |
| Argentina | Instituto Nacional de Estadística y Censos (INDEC) |
| Brazil | Instituto Brasileiro de Geografía e Estatistica (IBGE) |
| Chile | Instituto Nacional de Estadística (INE) |
| Colombia | Departamento Administrativo Nacional de Estadísticas (DANE). |
| Costa Rica | Instituto Nacional de Estadística y Censos Costa Rica (INEC). |
| El Salvador | Dirección General de Estadística y Censos (DIGESTYC)/SALURBAL |
| Mexico | Consejo Nacional de Población (CONAPO)/SALURBAL |
| Panama | Instituto Nacional de Estadística y Censos (INEC). |
| Peru | Instituto Nacional de Estadística e Informática (INEI) |

SALURAL = *Salud Urbana en América Latina*

Table S2: List of amenable causes of deaths and ICD codes:

| **Conditions** | | **Age range** | **ICD 10 codes** |
| --- | --- | --- | --- |
| Conditions for which health systems prevent incident cases | HIV/AIDS | 0-14 | B20-B24.9 |
|  | Vaccine preventable disease (including hepatitis B, otitis media, varicella, tetanus, diphtheria, measles, meningitis) | 0-74 | B16-B17.0, B19.1-B19.11, P35.3, A39-A39.4, A39.8-A39.9, A87-A87.9, D86.81, G00.0-G00.8, G03-G03.8, B01-B02.9, P35.8, A33-A35.0, A36-A36.9, B05-B05.9, A39-A39.4, A39.8-A39.9, A87-A87.9, D86.81, G00.0-G00.8, G03-G03.8 |
|  | Whooping cough | 0-4 | A37-A37.91 |
|  | Cervical cancer | 15-44 | C53-C53.9, D06-D06.9, D26.0 |
|  | Tuberculosis | 0-74 | A10-A14, A15-A19.9, B90-B90.9, K67.3, K93.0, M49.0, P37.0 |
| Acute conditions that are treated or cured with episodic care | Neglected tropical disease | 0-74 | B67-B67.4, B67.8-B67.99, B69-B69.9, B65-B65.9, A95-A95.9, B56-B56.9, B77-B77.9, B57-B57.5, B55.0, A90-A91.9, A83-A86.4, B94.1, F07.1, G04-G05.8 |
|  | Other infectious diseases (including malaria, intestinal infectious diseases, upper and lower respiratory infections) | 0-74 | B50-B53.8, A01.0-A01.4, A04.2, A04.4, A07.0-A07.1, A07.8-A07.9, A48.1, A70, J09-J15.8, J16-J16.9, J20-J21.9, P23.0-P23.4, J01-J01.91, J04.0, J05-J05.0, J05.11, J36-J36.0 |
|  | Diarrheal disease | 0-49 | A00-A00.9, A02-A04.1, A04.3, A04.5-A07, A07.2-A07.4, A08-A09.9, R19.7 |
|  | Maternal disorders | 15-44 | N96, N98-N98.9, O00-O07.9, O09-O16.9, O20-O26.93, O28- O36.93, O40-O48.1, O60-O77.9, O80-O92.79, O96-O99.91 |
|  | Neonatal disorders | 0-4 | P00-P04.2, P04.5-P05.9, P07-P15.9, P19-P22.9, P24-P29.9, P36- P36.9, P38-P39.9, P50-P61.9, P70.3-P72.9, P74-P78.9, P80- P81.9, P83-P84, P90-P94.9, P96, P96.3-P96.4, P96.8-P96.89 |
|  | Rheumatic heart disease | 0-44 | I01-I01.9, I02.0, I05-I09.9 |
|  | Ischemic heart disease | 0-74 | I20-I25.9 |
|  | Intracerebral hemorrhage | 0-74 | I60-I61.9, I62.0-I62.03, I67.0-I67.1, I68.1-I68.2, I69.0-I69.298 |
|  | Peptic ulcer disease | 0-74 | K25-K28.9, K31, K31.1-K31.6, K31.8, K31.82-K31.89 |
|  | Paralytic ileus and intestinal obstruction | 0-74 | K56-K56.9 |
|  | Appendicitis | 0-74 | K35-K37.9, K38.3-K38.9 |
|  | Inguinal, femoral, and abdominal hernia | 0-74 | K40-K42.9, K44-K46.9 |
|  | Gallbladder and biliary diseases | 0-74 | K80-K83.9 |
|  | Asthma | 0-14 | J45-J46.9 |
|  | Self-harm | 10-74 | X60-X84.9, Y87.0 |
|  | Road injuries | 0-74 | V01-V04.99, V06-V80.929, V82-V82.9, V87.2-V87.3 |
|  | Poisoning | 0-74 | X46-X47, X47.1-X47.8, X48-X48.9 |
|  | Adverse effect of medical treatment | 0-74 | Y38.9-Y84.9, Y88-Y88.3 |
| Chronic conditions requiring sustained care to either cure or prevent sequelae | HIV/AIDS | 15-74 | B20-B24.9 |
|  | Hypertensive heart disease | 0-74 | I11-I11.9 |
|  | Congenital heart anomalies | 0-14 | Q20-Q28.9 |
|  | Diabetes | 0-49 | E10-E10.11, E10.3-E11.1, E11.3-E12.1, E12.3-E13.11, E13.3- E14.1, E14.3-E14.9, P70.0-P70.2, R73-R73.9 |
|  | Breast cancer | 0-74 | C50-C50.929, D05-D05.92, D24-D24.9, D48.6-D48.62, D49.3, N60-N60.99 |
|  | Thyroid cancer | 0-74 | C73-C73.9, D09.3, D09.8, D34-D34.9, D44.0 |
|  | Colon and rectum cancer | 0-74 | C18-C21.9, D01.0-D01.3, D12-D12.9, D37.3-D37.5 |
|  | Uterine Cancer | 0-74 | C54-C54.9, D07.0-D07.2, N87-N87.9 |
|  | Malignant skin melanoma | 0-74 | C43-C43.9, D03-D03.9, D22-D23.9, D48.5 |
|  | Non-melanoma skin cancer | 0-74 | C44-C44.99, D04-D04.9, D49.2 |
|  | Testicular cancer | 0-74 | C62-C62.92, D29.2-D29.8, D40.1-D40.8 |
|  | Hodgkin lymphoma | 0-74 | C81-C81.99 |
|  | Leukemia | 0-74 | C91-C95.92 |
|  | COPD | 0-74 | J40-J44.9, J47-J47.9 |
|  | Chronic kidney disease (due to hypertension and due to diabetes) | 0-49 | I12-I13.9, E10.2-E10.29, E11.2-E11.29, E12.2, E13.2-E13.29, E14.2 |
|  | Epilepsy | 0-74 | G40-G41.9 |
|  | Alcohol use disorders | 15 -74 | F10-F10.99, G31.2, G72.1, P04.3, Q86.0, R78.0, X45-X45.9 |
|  | Drug use disorders | 15 -74 | F11-F16.99, F18-F19.99, P04.4-P04.49, P96.1, R78.1-R78.5 |

Figure S1: Completeness of death registration and % ill-defined deaths in 363 SALURBAL cities.

AR – Argentina, BR – Brazil, CL – Chile, CO – Colombia, CR Costa Rica, MX – Mexico, PA – Panama, PE – Peru, SV – El Salvador

Figure S2: Relationship between amenable mortality rates and population in 363 SALURBAL cities.

Table S3: Amenable mortality rate per 100,000, median, Q1, and Q3, by group in 363 SALURBAL cities by country.

|  |  | **Argentina** | **Brazil** | **Chile** | **Colombia** | **Costa Rica*** | **Mexico** | **Panama** | **Peru** | **El Salvador** |
| --- | --- | --- | --- | --- | --- | --- | --- | --- | --- | --- |
|  | Number of Cities | 33 | 152 | 21 | 35 | 1 | 92 | 3 | 23 | 3 |
| Female | Amenable deaths | 403.8 [374.1;431.6] | 520.1 [463.3;568.9] | 333.2 [313.2;356.7] | 514.6 [458.4;568.4] | 375.5 | 521.3 [453.4;576.9] | 370.7 [352.5;640.9] | 324.1 [272.4;387.4] | 282.2 [204.4;393.1] |
|  | Chronic | 193.4 [171.3;204.5] | 190.8 [171.5;209.2] | 150.5 [136.7;162.5] | 188.8 [178.3;210.1] | 161.5 | 204.8 [188.1;227.5] | 159.8 [151.1;219.1] | 131.8 [109.6;154.8] | 118.7 [90.4;124.8] |
|  | Acute | 188.6 [174.2;212.7] | 285.5 [255.9;323.2] | 166.6 [150;184.1] | 298.2 [250;322.5] | 196.0 | 284.5 [249.7;320.7] | 167.8 [163.6;264.7] | 150.3 [121.9;207.4] | 127 [99.6;234.2] |
|  | Preventable | 20.7 [18.1;26.7] | 33.1 [25.8;45.6] | 18.3 [14.3;21.6] | 40.1 [24;52] | 18.1 | 22.6 [15.1;33.9] | 51.8 [29.1;157.1] | 31.0 [24.3;48.5] | 34.1 [14.4;36.4] |
| Male | Amenable deaths | 664.4 [637;746.6] | 864.5 [800.9;964.5] | 589.6 [571.7;635.2] | 847.1 [734.4;959.1] | 592.1 | 920.6 [822.6;1014.7] | 668.5 [663.6;950.2] | 416.8 [358.5;490.4] | 501.7 [406.4;710.6] |
|  | Chronic | 192.4 [167.1;211.1] | 221.3 [196;244.9] | 142.1 [127.4;165.3] | 176.2 [158;202.1] | 140.6 | 236.4 [207.1;271.6] | 143.6 [142.2;198] | 97.8 [89.2;116.8] | 192.4 [142.1;269.8] |
|  | Acute | 453.4 [404.5;513.7] | 575.9 [515.5;651.5] | 402.8 [367.5;452.2] | 588.9 [500.4;657.3] | 411.3 | 595.0 [510.8;683.8] | 441.1 [380.2;509.7] | 252.1 [189;290.4] | 227.3 [220.9;358.9] |
|  | Preventable | 32.4 [23.4;40.6] | 63.8 [50;81.2] | 39.0 [34.7;45.9] | 94.4 [71.6;111.6] | 40.2 | 57.7 [37.8;90.8] | 146.1 [78.8;242.5] | 71.3 [47.7;86] | 81.9 [43.4;82] |

* Values are mortality rates for one city in Costa Rica

Figure S3: Estimates from final models (all cities) compared to models from sensitivity analysis that excluded cities in Peru and El Salvador.

PE – Peru, SV – El Salvador

SES – Socioeconomic
